# Oligonucleotides DNA containing 8-trifluoromethyl-2′-deoxyguanosine for observing Z-DNA structure

**DOI:** 10.1093/nar/gkaa505

**Published:** 2020-06-17

**Authors:** Hong-Liang Bao, Tatsuki Masuzawa, Takanori Oyoshi, Yan Xu

**Affiliations:** Division of Chemistry, Department of Medical Sciences, Faculty of Medicine, University of Miyazaki, 5200 Kihara, Kiyotake, Miyazaki 889-1692, Japan; Faculty of Science, Department of Chemistry, Shizuoka University, 836 Ohya Suruga Shizuoka 422-8529, Japan; Faculty of Science, Department of Chemistry, Shizuoka University, 836 Ohya Suruga Shizuoka 422-8529, Japan; Division of Chemistry, Department of Medical Sciences, Faculty of Medicine, University of Miyazaki, 5200 Kihara, Kiyotake, Miyazaki 889-1692, Japan

## Abstract

Z-DNA is known to be a left-handed alternative form of DNA and has important biological roles as well as being related to cancer and other genetic diseases. It is therefore important to investigate Z-DNA structure and related biological events in living cells. However, the development of molecular probes for the observation of Z-DNA structures inside living cells has not yet been realized. Here, we have succeeded in developing site-specific trifluoromethyl oligonucleotide DNA by incorporation of 8-trifluoromethyl-2′-deoxyguanosine (^F^G). 2D NMR strongly suggested that ^F^G adopted a syn conformation. Trifluoromethyl oligonucleotides dramatically stabilized Z-DNA, even under physiological salt concentrations. Furthermore, the trifluoromethyl DNA can be used to directly observe Z-form DNA structure and interaction of DNA with proteins *in vitro*, as well as in living human cells by^19^F NMR spectroscopy for the first time. These results provide valuable information to allow understanding of the structure and function of Z-DNA.

## INTRODUCTION

Z-DNA plays a critical role in gene expression (1), recombination (2,3), regulation (4,5). Previous evidence suggests that Z-DNA sequences are required for chromatin-dependent activation of the CSF1 promoter (6). A recent study showed that the Z-DNA binding domain of the vaccinia virus is necessary to inhibit IFN-primed virus-induced necroptosis (7). The amount of data that suggests the key role of Z-DNA in gene regulation continues to increase. Several proteins have been reported to specifically bind with Z-form DNA, such as ADAR1, DLM1, PKZ and E3L, and further regulate transcription or gene inhibition (8–20). Recent studies also suggested the relationship of Z-form structure and several diseases, such as cancer and inflammation (21–23). We recently suggested that influenza virus Z-form nucleic-acids induce ZBP1-mediated necroptosis (24). Therefore, the biological importance of Z-DNA lets it become attractive structures as a therapeutic target.

The investigation of the Z-DNA conformations associated with biological events is essential to understand the functions of Z-DNA. Despite the biological importance of the structure of Z-DNA, its chemical properties are not well understood, presumably because of the difficulty in obtaining stable Z-form oligonucleotides under physiological salt conditions (25–28), and the development of molecular probes for the observation of the structure of Z-DNA inside living cells has not yet been realized. To achieve this goal, there are two main challenges that need to be addressed: first, stabilizing the Z-DNA conformation under a physiological salt condition; secondly, a useful approach to investigate the Z-DNA structure in living cells.

In this study, we undertook the challenge of performing trifluoromethylation of 2′-deoxyguanosine-containing oligonucleotides and applying them to investigate Z-DNA structure and function. We succeeded in the one-step incorporation of a trifluoromethyl (CF_3_) group in the 8-position of 2′-deoxyguanosine and the site-specific incorporation of 8-trifluoromethyl-2′-deoxyguanosine (^F^G) into oligomer DNA sequences. The incorporation of CF_3_ would improve physicochemical properties and/or modulate the conformation of 2′-deoxyguanosine from *anti* to *syn* (29–32) and further dramatically stabilize the Z-DNA conformation at a physiological salt condition. Furthermore, after incorporation into biomolecules, the CF_3_ group can be used as a potential ^19^F NMR reporter tag for studying structures and properties of target molecules (33–42). ^19^F is an ideal conformational probe owing to its high sensitivity and low background signal in biological samples (35–42). We have recently shown that the ^19^F sensor can be used to study the G-quadruplex structure in living cells (38–40). The trifluoromethyl DNA can be used to directly study Z-form DNA structure and interaction of DNA with protein *in vitro*, as well as investigating the Z-DNA structure in living human cells by ^19^F NMR spectroscopy. Therefore, our developed trifluoromethyl oligonucleotide DNA perfectly addressed the two challenges above. These results provide valuable information to allow understanding of the structure and function of Z-DNA *in vitro* and in living cells.

## MATERIALS AND METHODS

### 8-trifluoromethyl-2′-deoxyguanosine

1.3 g (5.0 mmol) of 2′-deoxyguanosine was weighed and placed in a two-neck flask under argon. The following materials were added to the flask: 240 ml of dimethyl sulfoxide (DMSO), 7.5 ml of a 1 N sulfuric acid in DMSO, 5.0 ml of a 4.3 M DMSO solution of trifluoromethyl iodide (21.50 mmol), 0.17 ml of 30% hydrogen peroxide aqueous solution and 2.3 ml of a 1.0 M aqueous solution of iron (II) sulfate. The mixture was stirred for 15 min at room temperature. NaHCO_3_ aqueous solution was added to neutralize the mixture. The supernatant was obtained by centrifugation and extracted with 200 ml ethyl acetate. The water layer was evaporated and purification by silica gel column chromatography. 750 mg white solid product was obtained (yield: 45%). ^1^H NMR (400 MHz, DMSO-d6) δ 10.86 (s, 1H), 6.73 (m, 2H), 6.16 (t, *J* = 7.1 Hz, 1H), 5.29 (d, *J* = 4.3 Hz, 1H), 4.87 (t, *J* = 5.9 Hz, 1H), 4.43 (br s, 1H), 3.86 (m, 1H), 3.64 (m, 1H), 3.54 (m, 1H), 3.13 (m, 1H), 2.16 (q, *J* = 3.9 Hz,1H). ^13^C-NMR (100 MHz, DMSO-d6) δ 156.47, 154.29, 152,43, 132.77, 118.64, 116.46, 88.34, 84.67, 70.98, 61.92, 37.32. ^19^F NMR (372 MHz, DMSO-d6) δ 60.00. HRMS (ESI) for C_11_H_11_O_4_N_5_F_3_ [M−H]^−^: Calcd. 334.0758; Found. 334.0767.

### *N* ^2^-Dimethylformamidyl-8-trifluoromethyl-2′-deoxyguanosine (1)

^F^G (250 mg, 0.75 mmol) was co-evaporated with anhydrous DMF (5 ml) three times, followed by suspension in 4 ml of anhydrous DMF, and *N*,*N*-dimethylformamide diethyl acetal (0.9 ml, 5.3 mmol) was added. The mixture was stirred for at room temperature 1 h under argon. Then the solvent was evaporated and the residues were purified by medium pressure liquid chromatography (MPLC). Pale-yellow crystal of 1 (210 mg, 72%) was obtained. ^1^H NMR (400 MHz, DMSO-d6) δ 11.75 (s, 1H), 8.56 (s, 1H), 6.21 (t, *J* = 6.8 Hz, 1H), 5.38 (d, *J* = 6.8 Hz, 1H), 4.86 (t, *J* = 6.8 Hz, 1H), 4.50 (br s, 1H), 3.87 (m, 1H), 3.67 (m, 1H), 3.56 (m, 1H), 3.19 (s, 3H), 3.12 (m, 1H), 3.08 (s, 3H), 2.20 (m, 1H). HRMS (ESI) for C_14_H_16_O_6_N_4_F_3_ [M−H]^−^: Calcd. 389.1180; Found. 389.1186.

### 5′-*O*-Dimethoxytrityl-*N*^2^-dimethylformamidyl-8-trifluoromethyl-2′-deoxyguanosine (2)

Compound 2 (210 mg, 0.54 mmol) was dried three times with 5 ml of anhydrous pyridine and dissolved in anhydrous pyridine (6.5 ml). *N*,*N*-Diisopropylethylamine (0.36 ml, 2.16 mmol) and 4,4′-dimethoxytritylchloride (356 mg, 1.05 mmol) were added. The mixture was stirred at room temperature for 5 h under argon. Then 20 ml of CH_2_Cl_2_ and 20 ml of 5% NaHCO_3_ aqueous solution were added to stop the reaction. The water layer was extracted three times with CH_2_Cl_2_. The organic layer was dried over Na_2_SO_4_ and concentrated *in vacuo*. The residue was purified by MPLC to give compound 2 (295 mg, 79%) as a yellow solid. ^1^H NMR (400 MHz, CDCl_3_) δ 8.66 (s, 1H), 8.36 (s, 1H), 7.70–7.66 (m, 1H), 7.38–7.35 (m, 2H), 7.30–7.19 (m, 6H), 6.78–6.76 (m, 4H), 6.34 (t, *J* = 2.3 Hz, 1H), 4.79 (m, 1H), 3.95 (q, *J* = 5.8 Hz, 1H), 3.56 (s, 6H), 3.54 (t, *J* = 4.9 Hz, 1H), 3.31 (t, *J* = 3.6 Hz, 1H), 3.18 (q, *J* = 5.7 Hz, 1H), 3.07 (s, 3H), 2.95 (s, 3H), 2.38 (q, *J* = 5.5 Hz, 1H), 2.15 (d, *J* = 2.5 Hz, 1H). HRMS (ESI) for C_35_H_34_O_6_N_6_F_3_ [M−H]^−^: Calcd. 691.2486; Found. 691.2495.

### 3′-*O*-[(2-Cyanoethoxy)(diisopropylamino)phosphino]-5′-*O*-dimethoxytrityl-*N*^2^ dimethylformamidyl-8-trifluoromethyl-2′-deoxyguanosine (3)

Compound 3 (310 mg, 0.45 mmol) was dried three times by co-evaporation of 5 ml of anhydrous acetonitrile and dissolved in 5 ml of CH_2_Cl_2_. Then it was treated with dry *N*,*N*-diisopropylethylamine (291 μl, 1.8 mmol) and 2-cyanoethyl-*N*,*N*-diisopropylchlorophosphoramidite (390 μl, 1.8 mmol). The mixture was stirred at room temperature for 30 min under argon. After addition of 20 ml of CH_2_Cl_2_, the reaction was stopped by adding 20 ml of 5% NaHCO_3_ aqueous solution. The aqueous layer was extracted three times with CH_2_Cl_2_. The combined organic layers were dried over Na_2_SO_4_ and the solvent was evaporated *in vacuo*. The residue was purified by recycling HPLC to give compound 4 (320 mg, 80%) as a white solid. ^1^H NMR (400 MHz, CDCl_3_), 8.60 (s, 2H), 8.37 (s, 1H), 8.32 (s, 1H), 7.48 (m, 2H), 7.38–7.16 (m, 20H), 6.75–6.70 (m, 6H), 6.34–6.30 (m, 2H), 5.01 (q, *J* = 2.4 Hz, 1H), 4.87 (q, *J* = 3.7 Hz, 1H), 3.81–3.73 (m, 14H), 3.62–3.50 (m, 4H), 3.39–3.21 (m, 8H), 3.03 (s, 6H), 2.93 (m, 6H), 2.59–2.25 (m, 6H), 1.25–1.06 (m, 24H). ^19^F NMR (372 MHz, CDCl_3_) δ 60.88, 60.91. ^31^P NMR (161 MHz, CDCl3) δ 149.23, 148.97. HRMS (ESI) for C_44_H_51_O_7_N_8_F_3_P [M–H]^−^: Calcd. 891.3565; Found. 891.3578.

### DNA sample preparation

By using an automatic solid-phase phosphoramidite chemistry and DNA synthesizer, ^F^G was incorporated into the designed DNA sequence at a ratio of 1.0 μmol. After DNA synthesis, the ^F^G labelled DNA was cleavage from the column and deprotected by using AMA (Ammonium Hydroxide/40% aqueous methylamine 1:1 v/v) at room temperature for 20 min and at 338 K for 10 min, respectively. The oligomers were further purified by HPLC in a linear gradient of 50 mM ammonium formate in 1:1 acetonitrile/H_2_O and 50 mM ammonium formate in H_2_O. The oligomers were desalted through a NAP 10 column (GE Healthcare) and identified by MALDI-TOF-MS on an Autoflex III smart beam mass spectrometer (negative mode). For 6-mer 5′-CGC**^F^G**CG-3′ sequence, cal: 1858.33, found: 1859.21; for 8-mer C**^F^G**CAC**^F^G**CG sequence, cal: 2529.42, found: 2528.58.

### Zα protein preparation

The human ADAR1 plasmid was used as a template for polymerase chain reaction. The Zα domain (122–199) cDNAs of human ADAR1 protein was cloned into the pGEX-6p-1 vector (GE Healthcare, Chicago, IL, USA) between the EcoRI and XhoI sites using the following sets of primers to express an N-terminal glutathione S-transferase (GST) fusion protein: forward *d*(GCG GAT CCG GTG TTG ATT GCC TTT CCT CAC ATT), and reverse *d*(CGC TCG AGC TAG ACC GCG ATT TTC CAC AAA GGG GGT GTT). The construct was verified by automated DNA sequencing. DNA oligomers were obtained from Operon Biotechnologies (Japan). The *Escherichia coli* strain BL21 (DE3) pLysS-competent cells were transformed with the vector, and transformants were grown at 310 K in Luria Bertani medium containing ampicillin (0.1 mg/ml). Protein expression was induced at *A*_600_ = 0.6 with 0.1 mM isopropyl β-d-1-thiogalactopyranoside. Cells were harvested after 3 h and centrifuged at 6400 g for 20 min. The *E. coli* pellets were resuspended in 20 mM Tris–HCl (pH 8.0) containing 150 mM NaCl. The supernatants containing the expressed proteins were lysed by sonication (model UR-20P, Tomy Seiko, Japan) and centrifuged at 16 200 g for 15 min at 277 K. The supernatant and glutathione agarose (MilliporeSigma, Burlington, MA, USA) were incubated with gentle mixing for 1 h at 277 K; the resin was washed with 20 mM Tris–HCl (pH 8.0) containing 150 mM NaCl and 1% (v/v) Triton X-100) at 277 K. GST-tags were cleaved using a buffer containing 8 units/ml PreScission protease (GE Healthcare) on a resin for 16 h at 277 K, and the protein was eluted with 20 mM Tris–HCl (pH 8.0) containing 20 mM NaCl, 1 mM EDTA, and 1 mM dithiothreitol. Purification of protein was performed as described previously (43). The protein concentrations were determined using a BCA Protein Assay Kit (Thermo Scientific, Altham, MA, USA). The Zα protein was stocked with a final concentration in 350 μM with 20 mM Na-PO_4_ buffer (pH 7.0) and 1 mM DTT.

### CD measurement

CD experiments were performed by using a Jasco model J-820 CD spectrophotometer. For the salt concentration experiments, the Z-DNA sample was prepared at a 25 μM duplex concentration in the presence of 1 mM Na-PO_4_ buffer (pH 7.0) and various concentrations of sodium chloride. For DNA–protein binding experiment, the Z-DNA sample was prepared at the designed duplex concentration in the presence of 1 mM Na-PO_4_ buffer (pH 7.0) and different ratios of Zα protein.

### Introduction of trifluoromethyl DNA into HeLa cells by SLO treatment

The detailed procedure could refer to our previous report (40). HeLa cells (2 × 10^7^) grown in DMEM medium containing 10% FBS under a 5% CO_2_ atmosphere were collected and then washed twice with HBSS buffer. SLO (biologicalemia) was activated with 10 mM DTT and 0.05% BSA at 310 K for 2 h. To form pores in the plasma membrane, activated SLO was added to HeLa cells at a final concentration of 0.1 μg/ml, followed by gentle rotation incubation at 277 K for 15 min. After washing three times with ice-cold HBSS buffer, cells were incubated with 3 mM trifluoromethyl Z-DNA in 500 μl of HBSS buffer at 310 K for 30 min, and then shaken. Cells were resealed by adding ice-cold HBSS buffer (containing 1 mM CaCl_2_). After 30 min of incubation at 277 K, the cells were washed twice with HBSS buffer containing 1 mM CaCl_2_. The resealed cells were seeded in HBSS buffer containing 14% percoll (centrifuged at 2000 × g for about 1 h), and then centrifuged at 400 × g for 3 min. After centrifugation, the cell pellet (viable cells) was washed three times with HBSS buffer.

### ^19^F NMR measurement

Z-DNA samples were dissolved in 150 μl of a designed solution containing 1 mM Na-PO_4_ buffer (pH 7.0) and 10% D_2_O in various concentrations of NaCl. The ^19^F NMR spectrum was measured on a Bruker AVANCE 400 MHz spectrometer at a frequency of 376.05 MHz and referenced to the internal standard CF_3_COOH (–75.66 ppm). The experimental parameters are recorded as follows: spectral width 89.3 kHz, ^19^F excitation pulse 15.0 μs, relaxation delay 1.5 s, acquisition time 0.73 s, scan numbers 1024–4096, and line width 3. The ^19^F–^1^H HOESY experiment was performed on a Bruker AVANCE 400 MHz spectrometer using the hoesyfhqfqnrv pulse program at 296 K. Mixing time is 2 s. For DNA–protein binding experiment, the Z-DNA sample was prepared at 15 μM or 30 μM duplex concentration in the presence of 1 mM Na-PO_4_ buffer (pH 7.0) and different ratios of Zα protein. For in-cell ^19^F NMR measurement, the transfected cells were suspended in 200 μl of DMEM with 10% D_2_O and transferred to a Shigemi tube (Shigemi 5 mm Symmetrical NMR microtube). The experiment was performed at 296 K with a scan numbers value in 2048 or 4096. After the intracellular NMR measurement, 100 μl of DMEM was added to the cell suspension, and the supernatant was collected by centrifugation at 400 g for 3 min. The ^19^F NMR spectrum of the supernatant was measured with the same number of scans as the in-cell ^19^F NMR measurement.

### Molecular modeling

We manually generated the model of DNA structure based on the reported structure using the BIOVIA Discovery Studio 4.5. The molecular dynamics simulation was performed by the standard dynamics cascade in BIOVIA Discovery Studio 4.5 with some modifications. The structure was heated from 50 K to 300 K over 4 ps and equilibration at 300 K with 100 ps simulation time. The save results interval in the production step was 2 ps during 100 ps simulation time at 300 K. 10 best conformations generated by simulation were further energy minimized. The conformation with lowest energy was selected as shown in Figure 4.

## RESULTS AND DISCUSSION

### Synthesis of trifluoromethyl Z-DNA

**Scheme 1.**
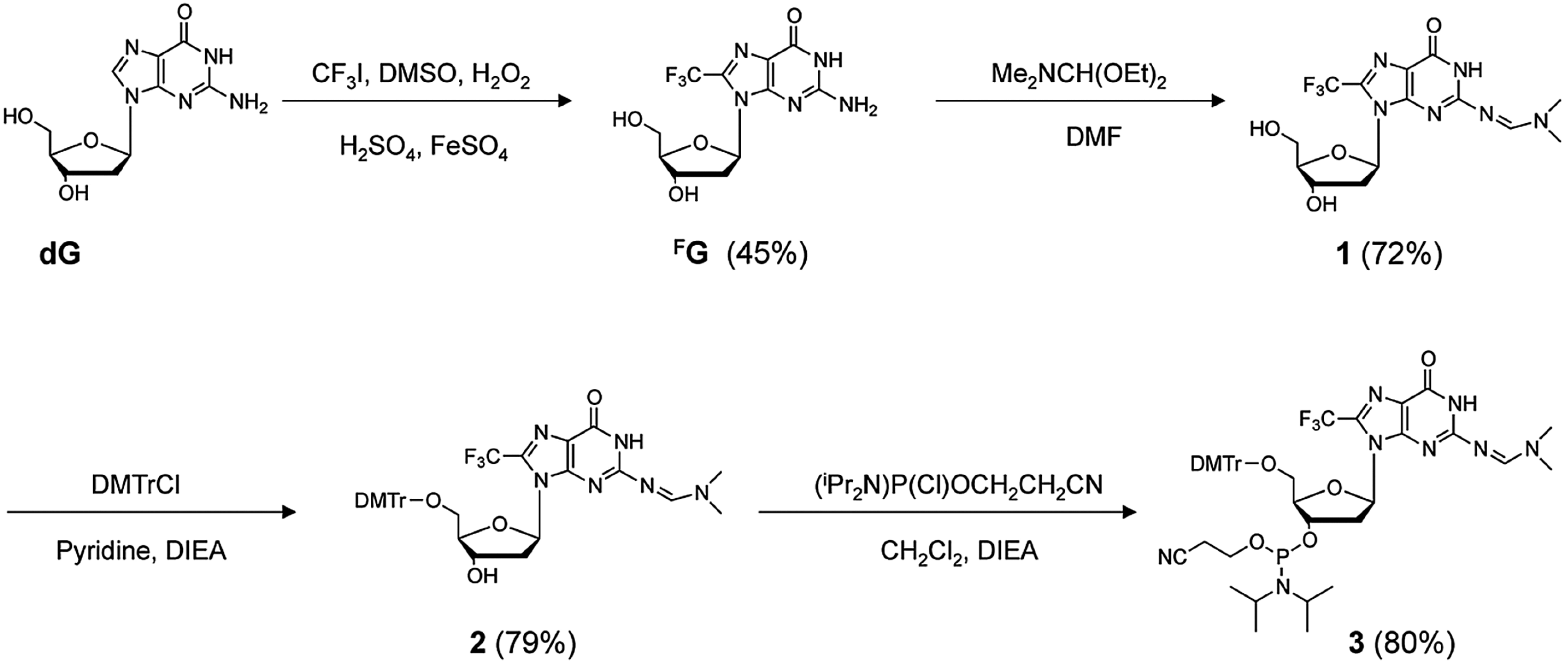
Synthesis of ^F^G and relative phosphoramidite compound.

**Figure 1.**
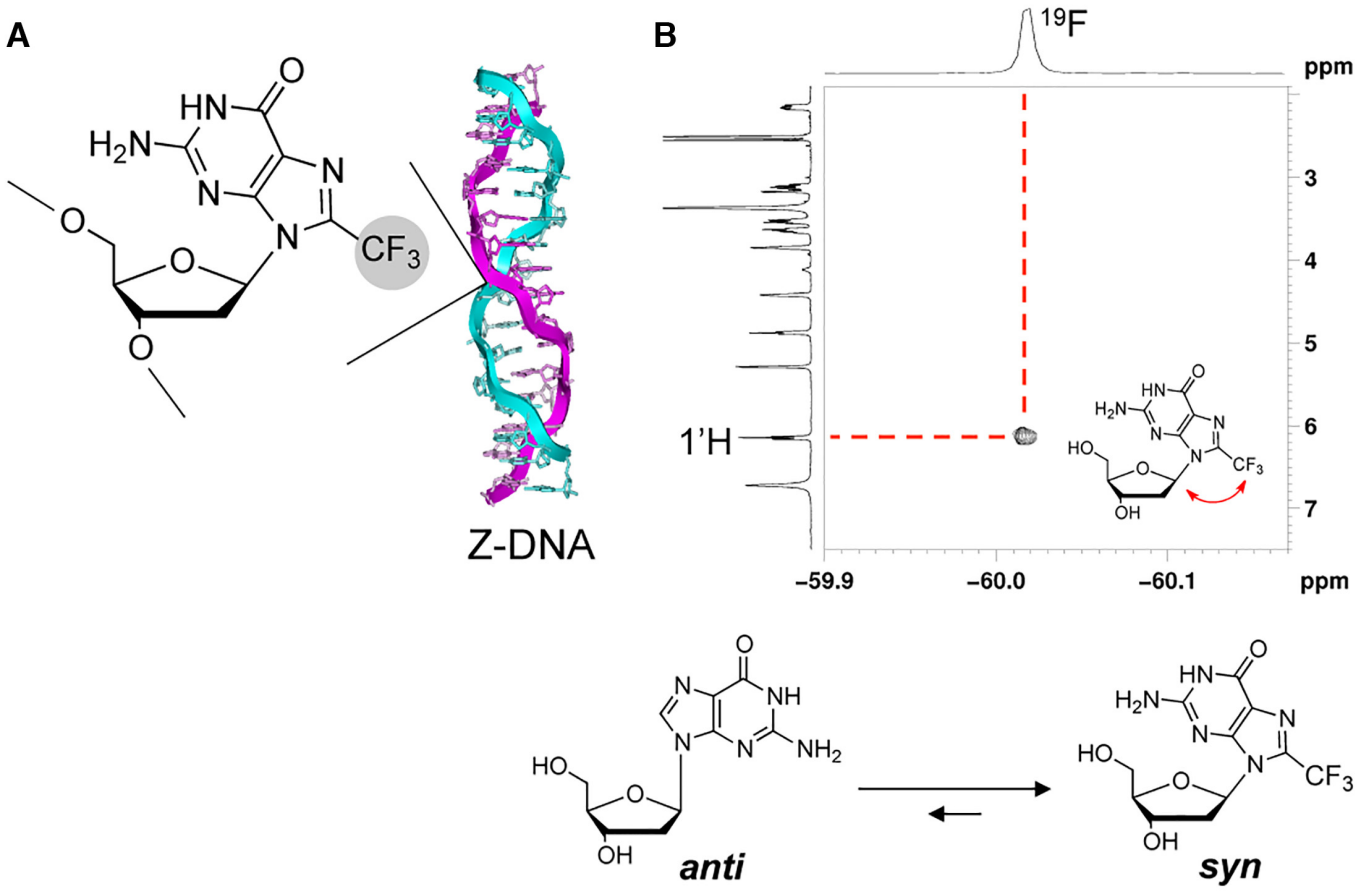
Schematic representation of trifluoromthyl Z-DNA and ^1^H-^19^F HOESY 2D spectrum. (**A**) Trifluoromethyl Z-DNA. (**B**) Chemical structure of ^F^G and ^1^H–^19^F HOESY 2D spectrum of ^F^G. The inset shows heteronuclear NOE of H of C1′ and ^19^F of CF_3_ with a red arrow. Incorporation of a CF_3_ group markedly stabilizes the *syn* conformation.

Using a catalytic system that consisted of H_2_O_2_, FeSO_4_ and H_2_SO_4_ in DMSO, we succeeded in the one-step synthesis of ^F^G through a radical reaction between dG and CF_3_I, with a yield of 45% (Scheme 1 and Supplementary Figure S1–S12). ^F^G is expected to adopt a *syn* conformation due to the space steric effect of the CF_3_ group at the 8-position of guanine. To verify our hypothesis, a ^19^F–^1^H heteronuclear Overhauser effect spectroscopy (HOESY) experiment was performed. The cross peak between the CF_3_ group at the C8 position and C1′H in the 2D NMR spectrum strongly suggested that ^F^G adopted a *syn* conformation (Scheme 1). This result is consistent with our previous reports that methylation was favorable for the *syn* conformation of the guanines (29–32). We further performed the site-specific incorporation of ^F^G into a series of DNA sequences by phosphoramidite chemistry (Figure 1).

### CD characterization of trifluoromethyl Z-DNA sequences

**Figure 2.**
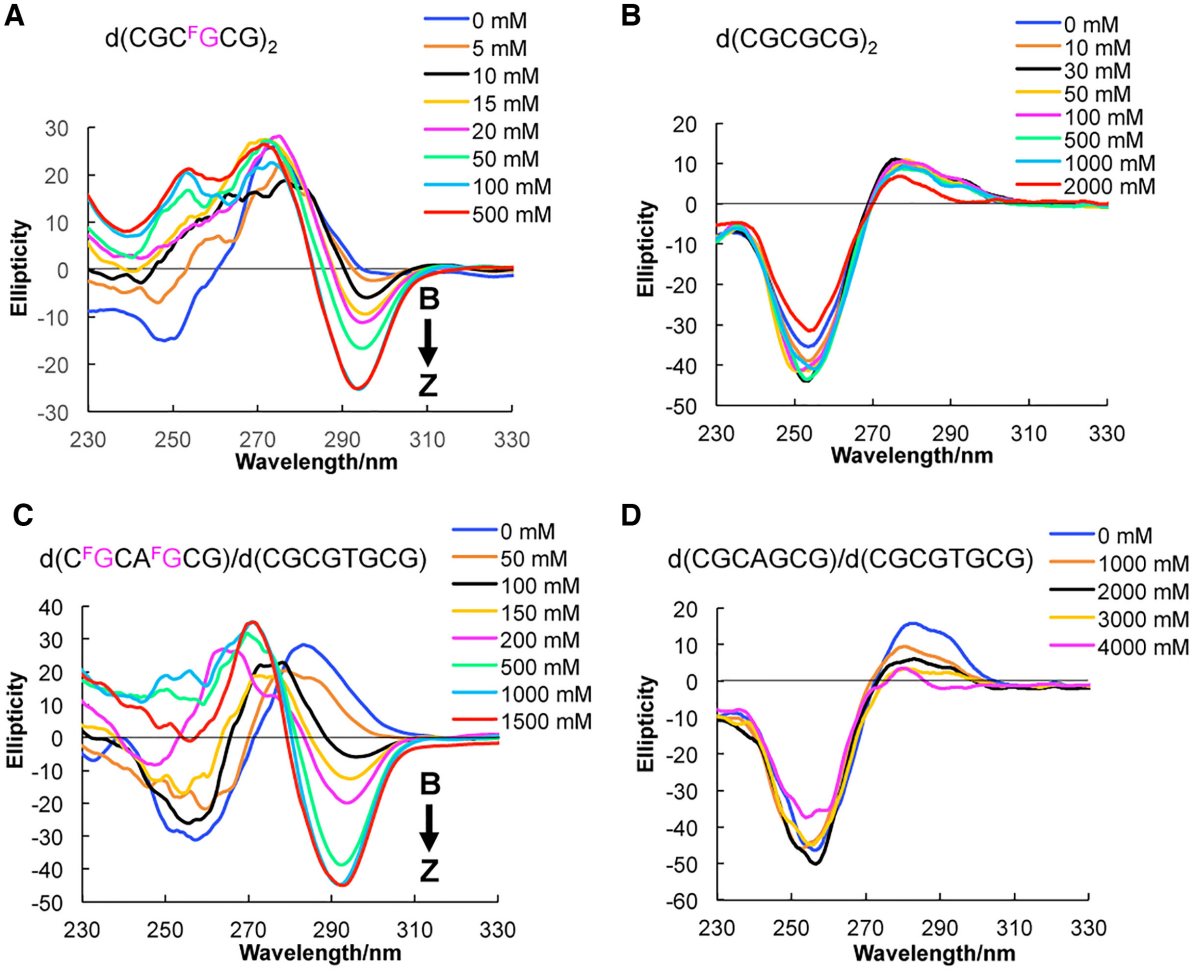
CD spectra of the B–Z transition at various NaCl concentrations. (**A**) CD spectra of trifluoromethyl DNA *d*(CGC^**F**^**G**CG)_2_, (**B**) Natural DNA d(CGCGCG)_2,_ (**C**) Trifluoromethyl DNA *d*(C^**F**^**G**CAC^**F**^**G**CG)/d(CGCGTGCG), and (**D**) Natural *d*(CGCACGCG)/d(CGCGTGCG) in 1 mM Na-PO_4_ buffer (pH 7.0), at 283 K. Various NaCl concentrations are indicated. ^F^G is colored in fuchsia.

Circular dichroism (CD) spectroscopy can be used to discriminate the B- and Z-DNA conformations (29–32,43). In Z-DNA, a negative Cotton effect appears at 295 nm, whereas in B-DNA, a more positive intense band appears at 280 nm. We used CD spectroscopy to monitor the conformational state at various NaCl concentrations (Figure 2). We observed that d(CGC**^F^G**CG)_2_ containing one ^F^G greatly stabilized the Z-DNA, showing a Z-form CD spectrum even in the presence of 10 mM NaCl at a lower physiological salt concentration (Figure 2A), and the midpoint for the B–Z transition was 22 mM (Table 1), while the value for the natural sequence was 2600 mM (Figure 2B and Table 1), which suggested that trifluoromethyl DNA could reduce the demand for NaCl by >100-fold compared with the natural sequence. The effect of Z-DNA stabilization was also confirmed in the 8-mer DNA duplex d(C**^F^G**CAC**^F^G**CG)/d(CGCGTGCG) with two ^F^G and an AT base pair that does not favor the formation of Z-DNA. The midpoint of the duplex with two ^F^G was the 148 mM NaCl concentration, which was lower than that of the natural 8-mer duplex (>5000 mM) (Figure 2C, D and Table 1). We further introduced the ^F^G into the complementary sequence of the 8-mer DNA sequence *d*(C**^F^G**CAC**^F^G**CG)/*d*(C**^F^G**CGT**^F^G**CG). The CD result suggested that even without addition of NaCl into the solution, the four positions modified DNA sequence can form a stable Z-DNA structure (Supplementary Figure S13). In Z-DNA, dG adopts a syn conformation; thus, the preferred syn conformation of ^F^G induced by the CF_3_ group contributed to the stabilization of the Z-DNA. Therefore, the CD experiment results suggested the ^F^G could dramatically stabilize the Z-DNA conformation at a physiological salt condition, which is quite important for further studying Z-DNA structure in living cells by ^19^F NMR spectroscopy. Other fluorinated nucleosides could be used as ^19^F NMR sensors (44), but they do not possess the ability to stabilize the Z-DNA conformation at physiological salt conditions. Therefore, this result highlights the superiority of ^F^G for further ^19^F NMR study of Z-DNA structure *in vitro* and in living cells compared with other fluorinated nucleosides.

**Table 1.** Midpoint NaCl concentrations for the B–Z transition in ^F^G modified Z-DNA sequences

Oligonucleotides	NaCl (mM)
*d*(CGCGCG)_2_	2600
*d*(CGC**^F^G**CG)_2_	22
*d*(CGCACGCG)/*d*(CGCGTGCG)	>5000
*d*(C**^F^G**CAC**^F^G**CG)/*d*(CGCGTGCG)	148
*d*(C**^F^G**CAC**^F^G**CG)/*d*(C**^F^G**CGT**^F^G**CG)	0

### 2D NMR analysis of trifluoromethyl Z-DNA structure

**Figure 3.**
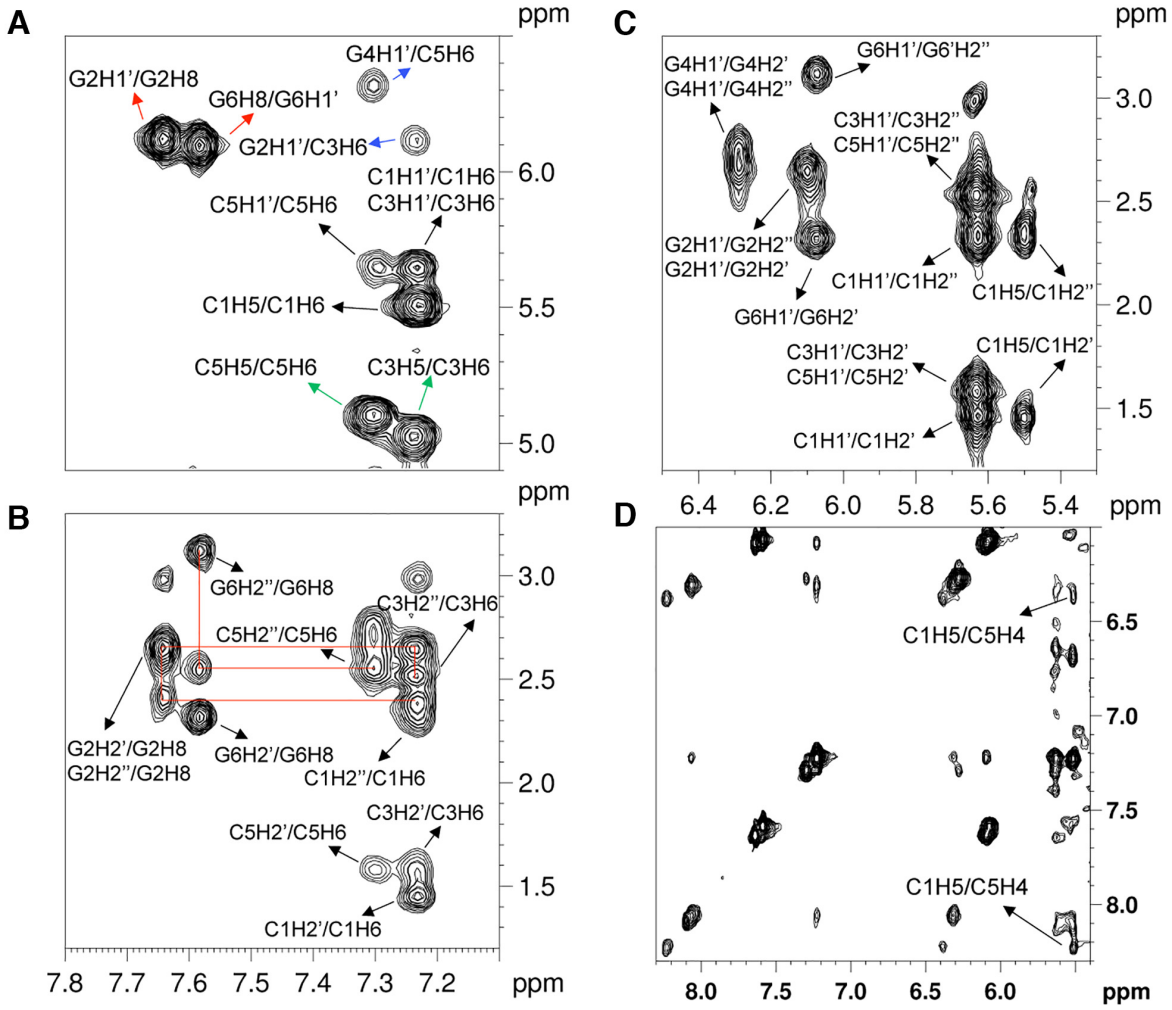
Z-DNA structural determination of *d*(C_1_G_2_C_3_**^F^G_4_**C_5_G_6_)_2_. (**A**) Aromatic-H1′/H5-H6 regions 2D NOESY spectrum of the modified *d*(CGC**^F^G**CG)_2_. Strong H8–H1′ cross peaks were observed (red arrows). C3H5 and C5H5 were fund to upshift 5.03 and 5.11 ppm (green arrows). The cross-peaks of internucleotide were marked with blue arrows. (**B**) Aromatic-H2′/H2″ regions 2D NOESY spectrum of the modified d(CGC**^F^G**CG)_2_. Sequential connections (red lines) were based on the aromatic-H2″ pathway. (**C**) H1′–H2′/H2″ regions 2D NOESY spectrum of the modified d(CGC**^F^G**CG)_2_. (**D**) The exchangeable proton 2D NOESY spectra of the H1′–aromatic region of *d*(CGC**^F^G**CG)_2_. There are clear NOE cross-peaks between C5H4 amino protons and C1H5 protons from the opposite strand.

We further performed the 2D NMR experiment to confirm the formation of Z-DNA structure of the trifluoromethyl modified DNA sequences in 100 mM NaCl. The non-exchangeable protons were assigned based on the 2D NOESY experiment in D_2_O (Figure 3). The H8/H1′ cross-peak region of the 2D NOESY spectrum showed only strong intranucleotide G2H8–G2H1′ and G6H8–G6H1′ cross-peaks, which indicated the *syn* conformation of dG residues (Figure 3A). Another evidence for the Z-DNA structure is the unusually upshift of the H5 proton of C3 and C5 to 5.03 and 5.11 ppm since the ring current influence of the dG bases on the 3′-side in Z-DNA (28). We further extended the assignment to the aromatic-H2′/H2″ (Figure 3B) and finally to all regions of the spectrum. Sequential assignments of C1 to C3 and G4 to G6 for the Z-DNA can be detected based on the aromatic-H2″ pathway (Figure 3B, red lines), indicating the sequence-specific connectivity for left-handed helices. Figure 3C showed clearly cross-peaks between H1′ and H2′/H2″. The chemical shifts of all ^1^H signals are displayed in Supplementary Table S1. Consistent with previous studies, the H2′ and H5′ proton of dC unusually upfield also, indicated the Z-DNA conformation (28).

The exchangeable proton NMR spectrum in 90% H_2_O of the modified sequence was shown in Figure 3D and Supplementary Figure S14. The cross-peaks of the exchangeable protons are also consistent with Z-DNA conformation. Similar to the previous study, the cross-peaks of C5H4 (amino) and C1H5 were observed (Figure 3D) (28). These cross-peaks could be only observed in the Z-DNA conformation. Moreover, the clear cross-peaks of the imino proton of dG (∼13.3 ppm) and the amino proton of dC suggested Watson–Crick base pairs (Supplementary Figure S14). All of the evidence suggested the ^F^G modified 6-mer oligonucleotide could adopt Z-DNA structure under a physiological salt condition.

**Figure 4.**
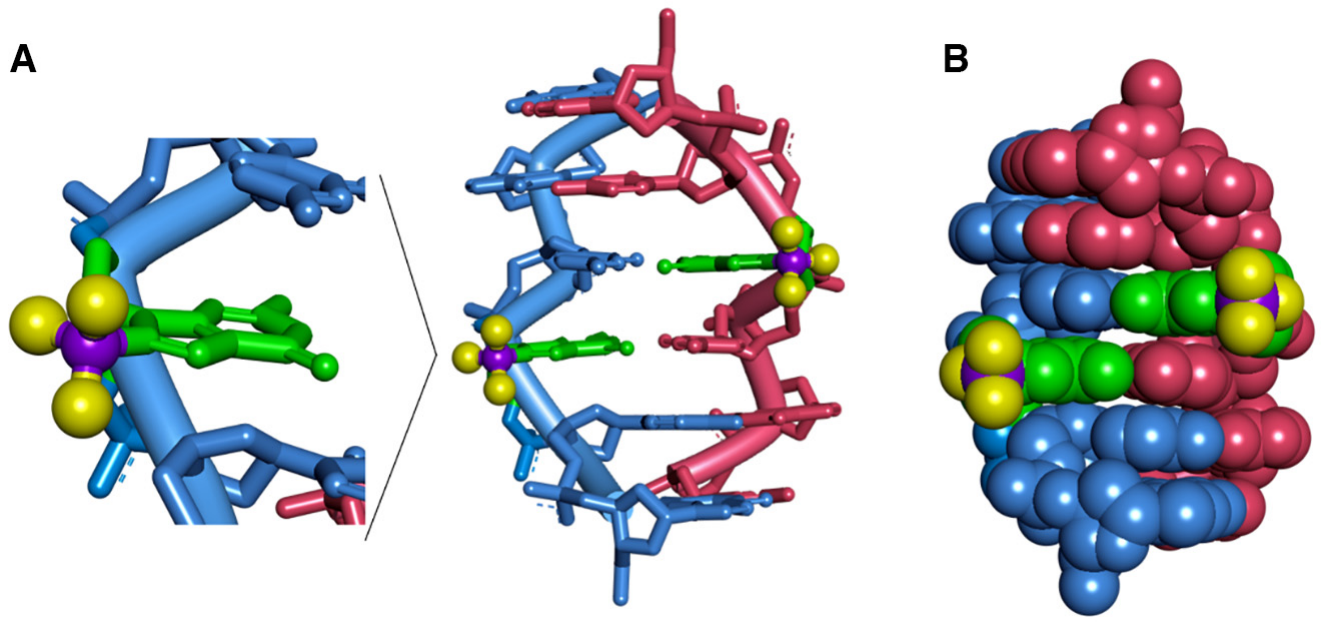
The structural model for d(CGC**^F^G**CG)_2_ Z-DNA. (**A**) ^F^G is expanded in green and the fluorine and carbon of C8-trifluoromethyl are colored with yellow and purple, respectively. (**B**) Ball and stick view of the Z-DNA structure. C8-trifluoromethyl groups were located outside of Z-DNA.

The structural model of d(CGC**^F^G**CG)_2_ is constructed based on the reported Z-form structure and NOE-constrained refining method (31). The molecular dynamics simulation was carried out in BIOVIA Discovery Studio 4.5 through a standard dynamic cascade with some modifications. The lowest energy conformation was selected as shown in Figure 4. The hydrophobic trifluoromethyl groups are located outside of the helix, which is consistent with the previous studies that the induction of a trifluoromethyl group into the C8 position of dG could strongly improve the stability of Z-form oligonucleotide.

### ^19^F NMR characterization of trifluoromethyl Z-DNA structure *in vitro* and in living Hela cells

**Figure 5.**
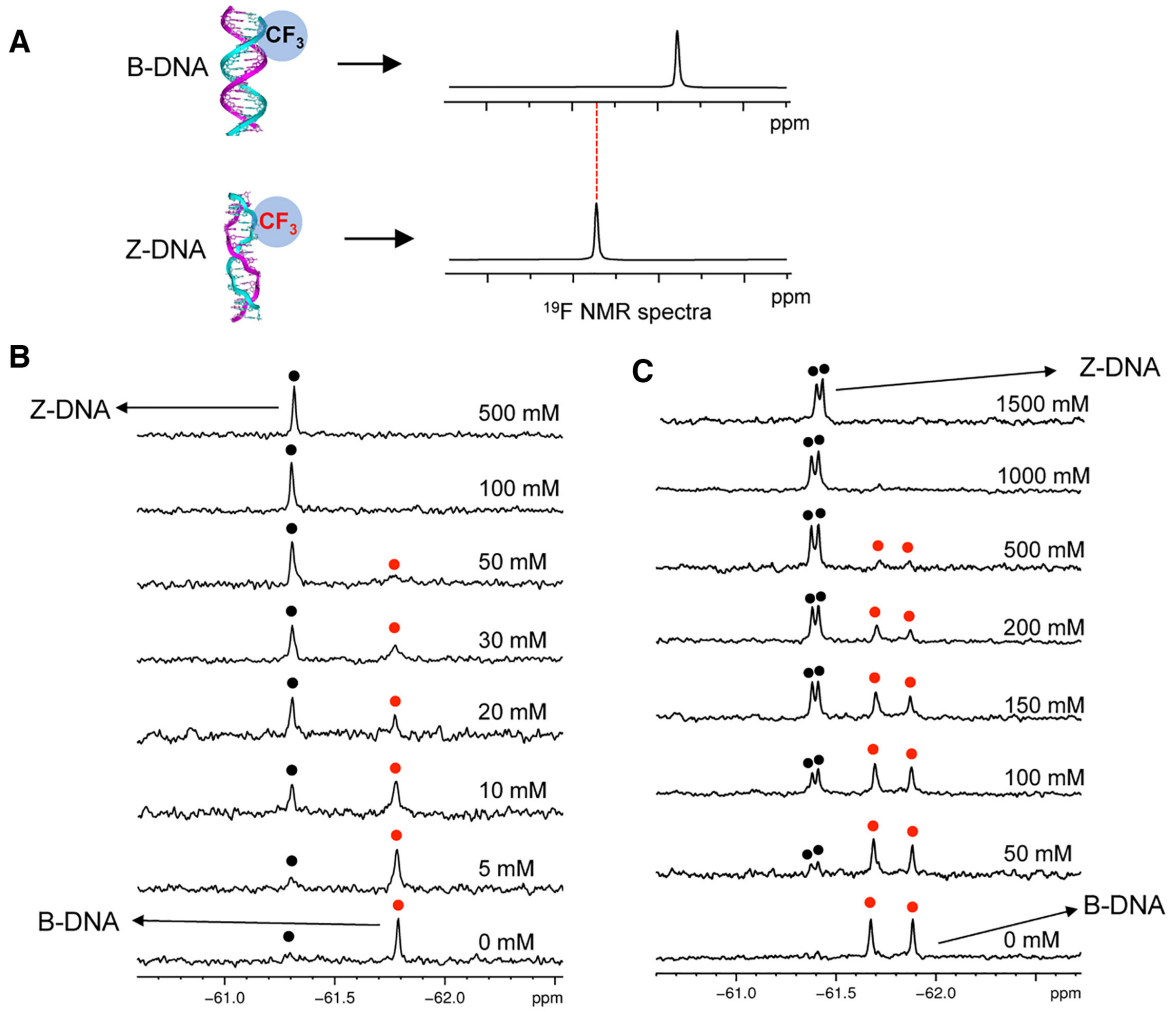
^19^F NMR experiments for study B-Z transition in *vitro*. (**A**) Concept for the detection of B–Z transition by ^19^F NMR. Two ^19^F resonances of different chemical shifts are expected according to B-DNA and Z-DNA. (**B**) ^19^F NMR spectra of d(CGC**^F^G**CG)_2_ in 1 mM Na-PO_4_ buffer (pH 7.0) and various NaCl concentrations. (**C**) ^19^F NMR spectra of *d*(C**^F^G**CAC**^F^G**CG)/d(CGCGTGCG) in 1 mM Na-PO_4_ buffer (pH 7.0) and various NaCl concentrations. The ^19^F NMR spectra were recorded at 50 μM duplex concentration. Red and black spots indicated B-form and Z-form DNA, respectively.

Based on the concept that ^19^F NMR signals are strongly dependent on the structural environment of the ^19^F label, the ^19^F atom in trifluoromethyl DNA as a ^19^F sensor provides the ^19^F NMR signals that can be used as a powerful tool for the analysis of biomolecule conformation by ^19^F NMR spectroscopy. Thus, it is possible to distinguish B- and Z-DNA structures by the different chemical shifts in ^19^F NMR spectra (Figure 5A). In 6-mer duplex DNA d(CGC**^F^G**CG)_2_, only one signal was observed at −61.79 ppm in the absence of NaCl, indicating that the B-form DNA was consistent with the CD result. A new signal appears as the NaCl concentration increases (−61.29 ppm). The new signal is clearly observed at a NaCl concentration of 10 mM. The intensity of the new signal is markedly greater than that of the initial peak at 30 mM NaCl concentration (Figure 5B). To combine the CD and 2D NOESY results, we assigned the new peak as a Z-DNA structure. The 8-mer DNA duplex with two ^F^G was then created to confirm the assignment using ^19^F NMR (Figure 5C). There are two ^19^F NMR signals could be observed in the absence of NaCl. Two ^19^F NMR peaks result from two asymmetric ^F^G due to their different positions within the 8-mer duplex sequence. With increasing NaCl concentration, the two peaks significantly decreased and completely disappeared in B-DNA, and two new strong-intensity peaks appeared as Z-DNA. Thus, we could conclude that the trifluoromethylated oligonucleotide DNA allowed us to directly observe the DNA structural conversion from B- to Z-DNA. The ^19^F NMR experiment was also performed with the four positions modified 8-mer DNA (Supplementary Figure S13b). Four ^19^F NMR signals were observed between –61.3 and –61.5 ppm, which are assigned to the four modified dGs. There is no chemical shift change after addition of NaCl into this sample. This ^19^F NMR result is in good agreement with the CD spectra that the four positions modified sequence could form a stable Z-DNA structure even in the absence of NaCl and further addition of NaCl could not change the DNA conformation.

**Figure 6.**
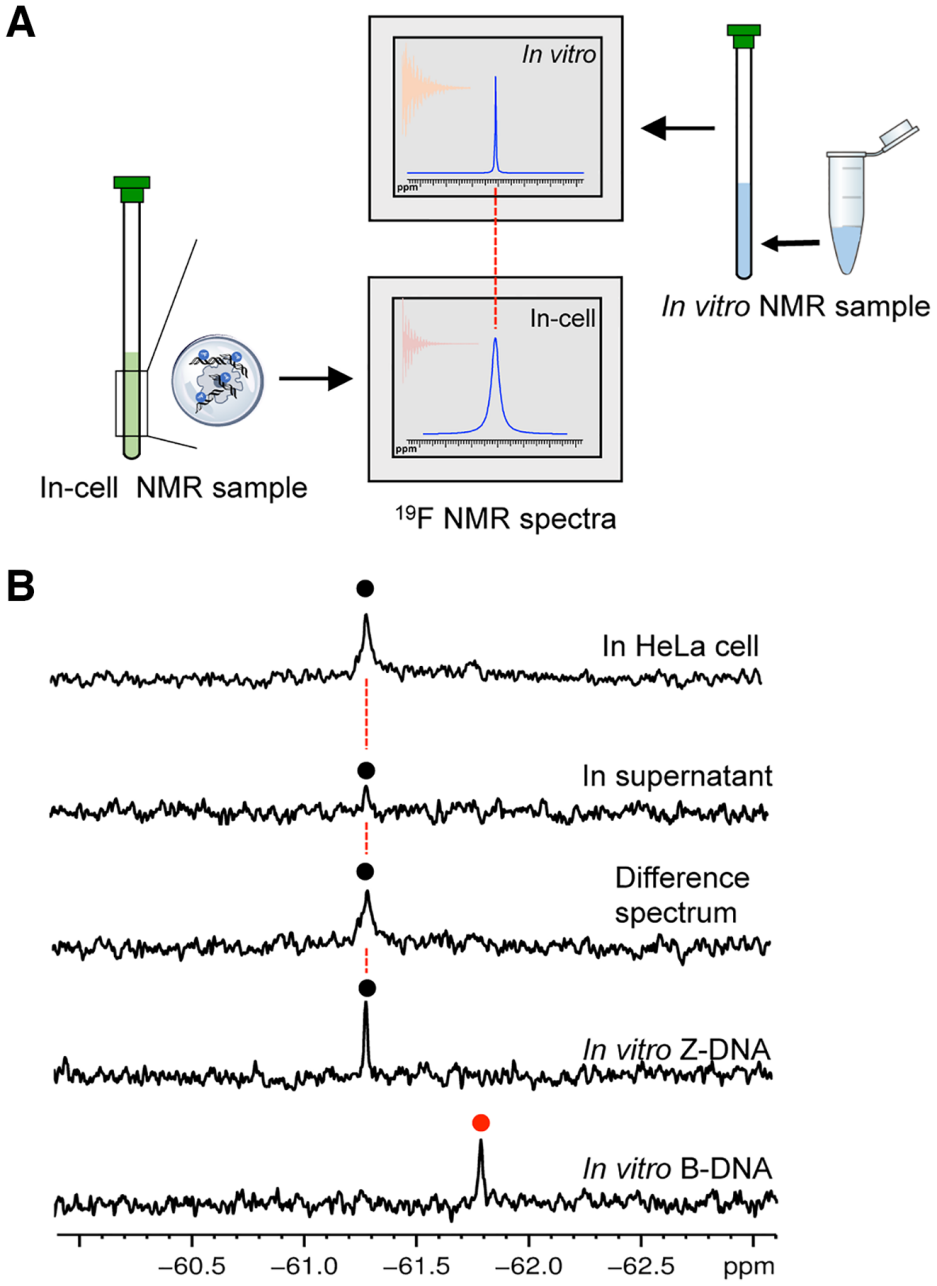
Schematic overview of in-cell Z-DNA ^19^F NMR experiments. (**A**) The comparison with the position of reference *in vitro* spectrum provides a reliable determination of intracellular Z-DNA. (**B**) Comparison of ^19^F NMR spectra for *in vitro* B-DNA, *in vitro* Z-DNA, in HeLa cell, in supernatant and difference spectrum between HeLa cell and supernatant.

Encouraged by the ability to use ^19^F NMR spectroscopy to monitor the conformation of DNA, we utilized ^19^F NMR to observe Z-DNA in living human cells. In-cell NMR is a powerful tool to study the structure and relative biological event at the really intracellular environment (45,46). Direct observation of the Z-DNA structure in human cells will offer critical information to further understand its biological functions. An in-cell ^19^F NMR spectroscopy strategy is shown in Figure 6A, showing that comparing the in-cell ^19^F-NMR spectrum to the *in vitro* result as a reference enables a reliable determination of the intracellular Z-DNA conformation. The d(CGC**^F^G**CG)_2_ sequence was transfected into HeLa cells by using an SLO treatment approach (40). Figure 6B shows a comparison of the *in vitro* B-DNA, Z-DNA and in-cell NMR spectra. One signal was observed at the bottom of the in-cell NMR spectrum, for which the chemical shift was identical to that observed for the corresponding Z-DNA in the *in vitro*^19^F NMR spectrum (top). The *in vivo* NMR spectrum has a little broad line width compared to the sample in dilute solution due to the inherent sample inhomogeneity and high viscosity of the intracellular environment (47). After the NMR measurement, the suspension was collected and examined by ^19^F NMR spectroscopy. Almost no signal was observed from the supernatant (Figure 6B), indicating that almost all of the NMR signals originated from the ^19^F labeled DNA within the HeLa cells. We also generated a difference spectrum between the HeLa cells and the suspension to eliminate the signal from the supernatant. Thus, the in-cell ^19^F NMR data demonstrate that the trifluoromethyl DNA can be present in a Z-DNA conformation in living cells. To our best knowledge, this is the first time to directly observe the Z-DNA conformation in living cells by NMR spectroscopy.

We further performed the in-cell ^19^F NMR experiment of the modified 8-mer DNA sequence *d*(C**^F^G**CAC**^F^G**CG)/d(CGCGTGCG), which requires a higher concentration of NaCl for B–Z transition. As shown in Supplementary Figure S15, the 8-mer DNA sequence could form both Z-DNA and B-DNA structure in Hela cells. This result could offer the opportunity for further investigating the B-Z DNA structural transition in living cells in the future.

We further tried to estimate the effective concentration of the trifluoromethyl DNA within cells. It is difficult to directly obtain the transfected DNA concentration in living cells. In a previous study, the authors used the same SLO approach to transfect protein into HeLa cells for in-cell NMR study (48). They demonstrated that 1 mM protein incubated with cells could result in 50 μM intracellular concentration of protein and further yield an estimate of 4 μM for the protein concentration of the NMR sample. In this study, we used the same approach to transfect the DNA into Hela cells and a 3 mM concentration of DNA was used to incubate with the cells. Therefore, we assumed that approximately 150 μM transfected DNA concentration could be obtained and the final concentration of the NMR sample is about 12 μM. To verify the speculate, we further performed the *in vitro* experiment of the trifluoromethyl 6-mer DNA sample at 12 μM strand concentration to compare with the in-cell spectrum (Figure 6B, bottom). We found that the intensity of the *in vitro* sample is comparable with the in-cell result. Therefore, we demonstrated that the transfect DNA concentration in living cells is about 150 μM.

### Monitor DNA-protein interaction by ^19^F NMR spectroscopy

**Figure 7.**
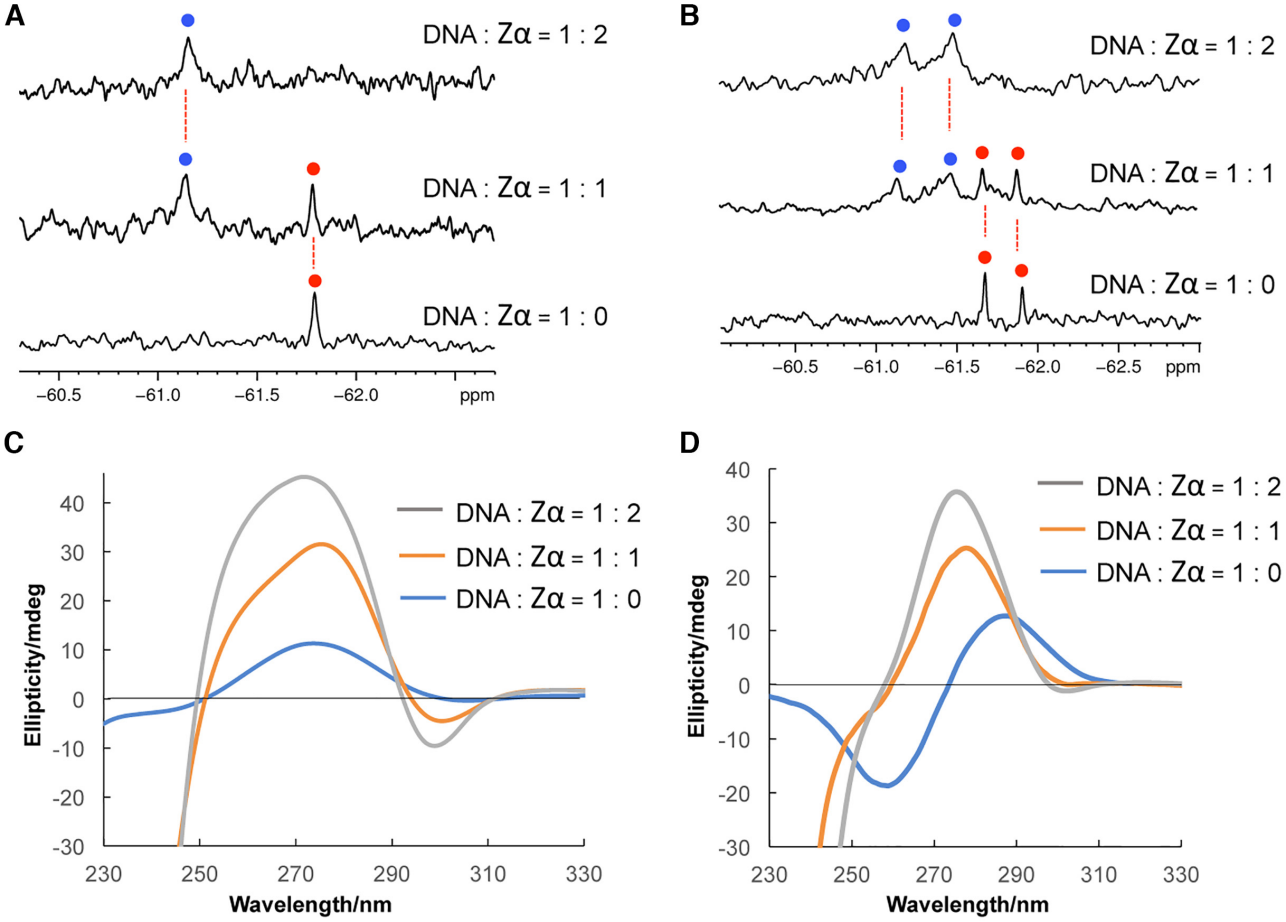
Characterization of DNA–protein interaction. (**A**) ^19^F NMR spectra of 5′-CGC**^F^G**CG-3′ in the presence of Zα with (0, 1, 2 equiv) as indicated on the right. (**B**) ^19^F NMR spectra of DNA *d*(C**^F^G**CAC**^F^G**CG)/*d*(CGCGTGCG) in the presence of Zα with (0, 1, 2 equiv) as indicated on the right. Conditions: [duplex DNA] = 15 μM, [Na-PO_4_] = 1 mM (pH 7.0), and 1 mM DTT. (**C**) CD spectra of 5′-CGC**^F^G**CG-3′ and Zα in the presence of Zα with (0, 1, 2 equiv) as indicated on the right. (**D**) CD spectra of *d*(C**^F^G**CAC**^F^G**CG)/*d*(CGCGTGCG) and Zα in the presence of Zα with (0, 1, 2 equiv) as indicated on the right. Conditions: [duplex DNA] = 15 μM, [Na-PO_4_] = 1 mM (pH 7.0) and 1 mM DTT.

Finally, we utilized the trifluoromethyl oligonucleotide to study DNA–protein interactions. Zα domain, an NH_2_-terminus of ADAR1, is reported for binding to Z-form DNA with high-affinity and stabilizing the Z-DNA structure (49). When the Zα binds to the DNA, a different ^19^F NMR chemical shift is expected to be observed because the local environment around the trifluoromethyl group changed. Addition of the 1 equivalent Zα to a solution of ^F^G labeled 6-mer DNA sample induced a new signal at –61.13 ppm in the ^19^F NMR spectra (Figure 7A). The new signal was assigned to the complex of DNA-Zα in accordance with the previous reports (11,43). We note that the original signal of free DNA still remains, which suggested 1 equivalent Zα is not enough to bind with the 6-mer DNA. When 2 equivalent Zα domain was added to the DNA sample, the original peak for the free DNA disappeared and only the complex of DNA-Zα signal appeared, which is in good agreement with previous studies that a Z-DNA sequence could bind with two Zα (11,43). We also performed the ^19^F NMR experiment to monitor the interaction of 8-mer DNA *d*(C**^F^G**CAC**^F^G**CG)/*d*(CGCGTGCG) and Zα protein (Figure 7B). After addition of 1 equivalent Zα, two new peaks appeared and the original peaks for free DNA completely disappeared after the addition of 2 equivalent Zα protein. CD experiment was employed to verify the binding of Zα and ^F^G labeled DNA sequences (Figure 7C and D). In the 1: 1 ratio of DNA and Zα, the peak intensity at 295 nm decreased and around 275 nm increased, which indicated the Zα binds with ^F^G labeled 6-mer and 8-mer DNA and stabilizes the Z-DNA conformation, consistent with our previous report (43). Addition of 2 equivalent Zα could further change the intensity of the peaks. These results demonstrate that the trifluoromethyl DNA can be used to investigate the interaction of Z-DNA with protein. We further performed ^19^F NMR and CD experiments of the four positions modified DNA *d*(C^**F**^**G**CAC^**F**^**G**CG)/*d*(C^**F**^**G**CGT^**F**^**G**CG) and Zα protein (Supplementary Figure S16). In the ^19^F NMR spectra, the four peaks for Z-DNA conformation disappeared after addition of Zα protein and a broad signal appeared, which indicated the binding of Z-DNA and Zα protein. In the CD spectra, the addition of Zα protein did not induce a big change compared with the two positions modified 8-mer DNA (Figure 7D), because the four positions modified 8-mer sequence can form Z-DNA structure even in the absence of NaCl or Zα protein. We also employed the CD experiment to monitor the interaction of native 8-mer DNA *d*(CGCACCG)/*d*(CGCGTGCG) and Zα protein (Supplementary Figure S17). The CD result indicated that Zα protein could induce the B–Z transition of the native 8-mer DNA, but transition is not particularly effective in comparison with the two positions modified 8-mer DNA (Figure 7D), since the intensity of the peak around 275 nm for the modified sequence is about two times stronger than the native 8-mer DNA. We also found that the Zα protein alone does not show the signals between 250 and 320 nm (Supplementary Figure S18).

In conclusion, we have succeeded in incorporating a CF_3_ group into the 8-position of 2′-deoxyguanosine and site-specific trifluoromethyl oligonucleotide DNA. The trifluoromethyl oligonucleotides markedly stabilized the Z-DNA even under physiological salt concentrations. Furthermore, the CF_3_ group can be used as a ^19^F NMR probe to study Z-DNA structure and DNA–protein binding reaction *in vitro* and investigate the Z-DNA structure in living cells by ^19^F NMR spectroscopy. The results demonstrated that trifluoromethyl oligonucleotide DNA provides a new approach for Z-DNA structure and function studies.

## Supplementary Material

gkaa505_Supplemental_File
