# Supplementary material for: Oligonucleotides DNA containing 8-trifluoromethyl-2′-deoxyguanosine for observing Z-DNA structure
Source: Nucleic Acids Res. 2020 Jun 17;48(13):7041–51. doi: 10.1093/nar/gkaa505 (PMC7367190; doi:10.1093/nar/gkaa505)
Supplement: gkaa505_Supplemental_File [file gkaa505_supplemental_file.pdf]

Supporting Information

General.

^{1}H-NMR, ^{13}C-NMR, ^{19}F-NMR and ^{31}P-NMR spectra were recorded on a BRUKER (AV-400M) magnetic resonance spectrometer. DMSO-d6 and $CDCl_3$ were used as the solvents. Coupling constants (J) values are given in Hz and are correct to within 0.5 Hz. Signal patterns are indicated as br, broad; s, singlet; d, doublet; t, triplet; m, multiplet. All reagents were purchased from Aldrich, TCI (Tokyo Chemical Industry Co., Ltd.) or Wako (Wako Pure Chemical Industries, Ltd.). Thin layer chromatography was performed using TLC Silica gel 60 F254 (Merck). Compounds were visualized by staining with a potassium permanganate solution. High-resolution mass spectra (HRMS) were recorded by electrospray ionization (ESI) on a Thermo Scientific Q Exactive instrument.

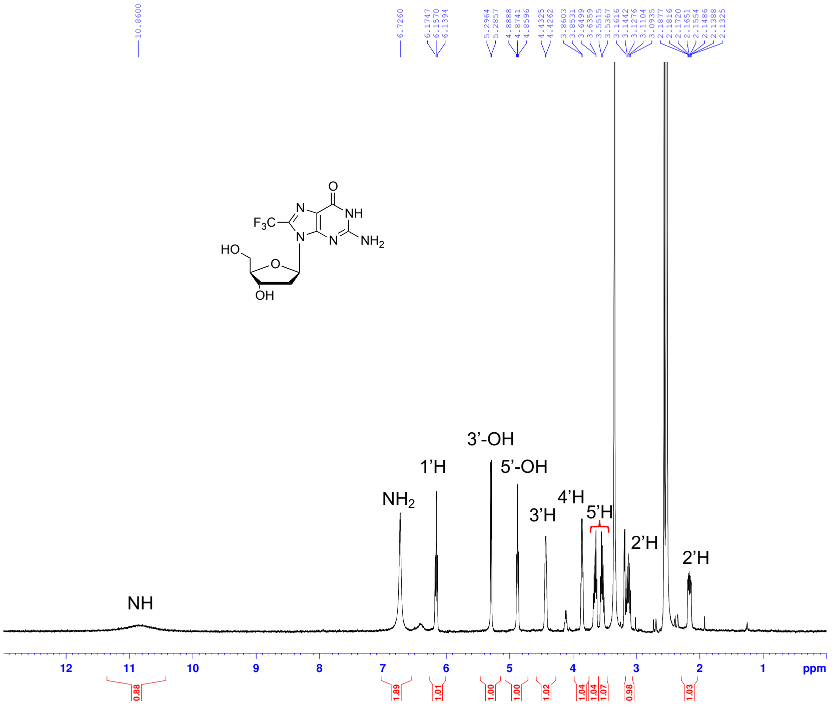

Supplementary Figure S1. ^{1}H NMR spectrum of ^{F}G.

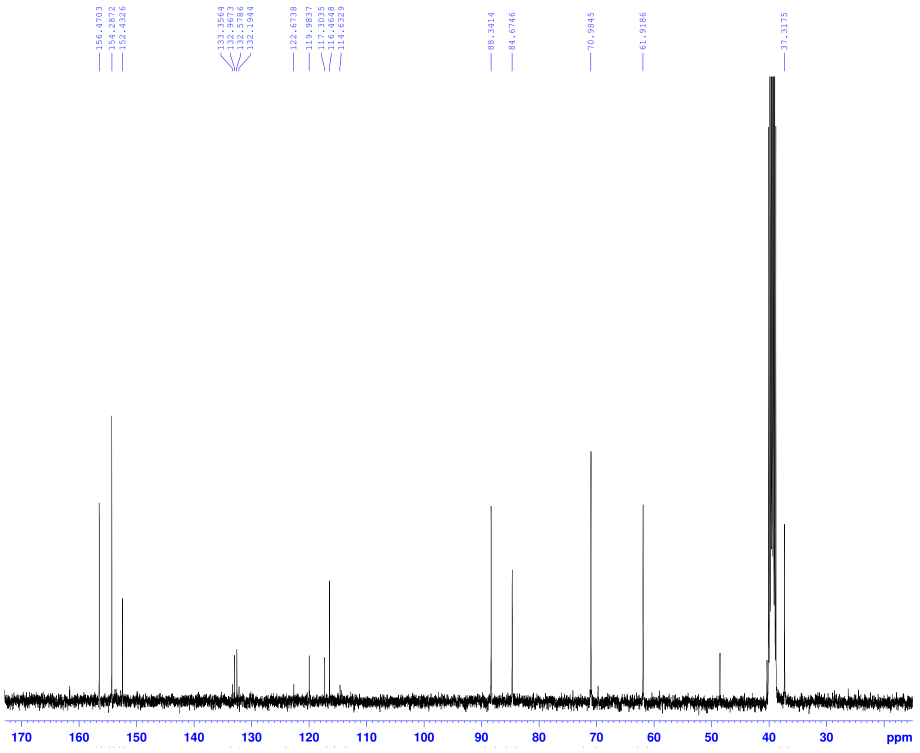

Supplementary Figure S2. ^{13}C NMR spectrum of ^{F}G.

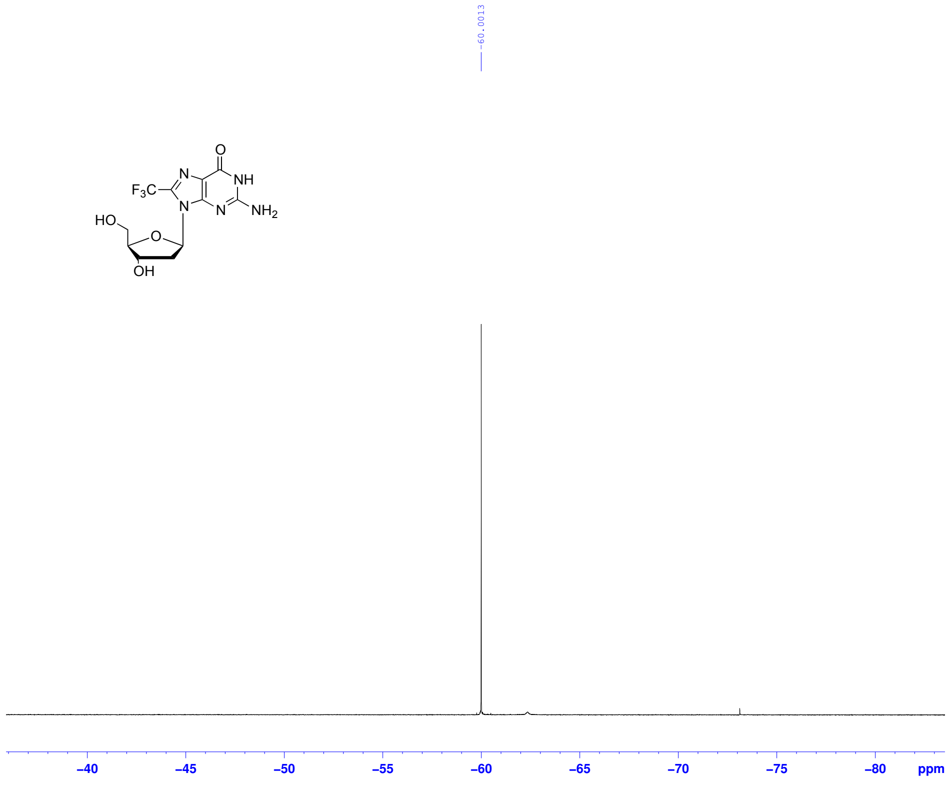

Supplementary Figure S3. ^{19}F NMR spectrum of ^{F}G.

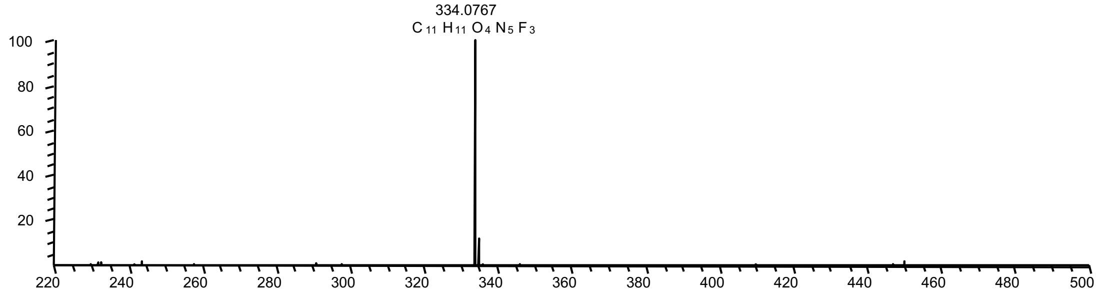

Supplementary Figure S4. HRMS spectrum of ^{F}G.

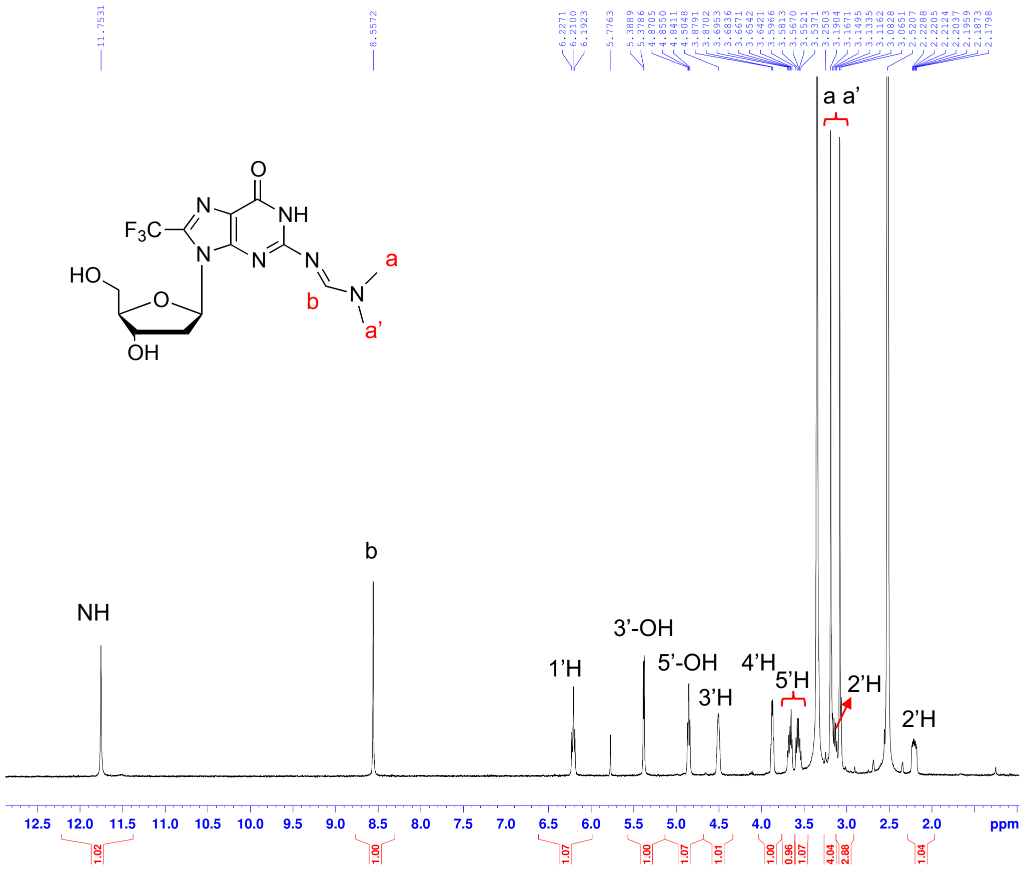

Supplementary Figure S5. ^{1}H NMR spectrum of compound 1

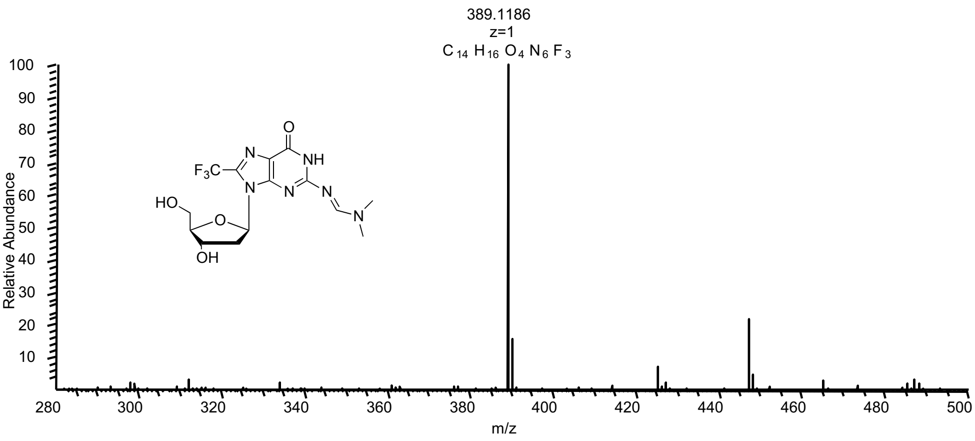

Supplementary Figure S6. HRMS spectrum of compound 1

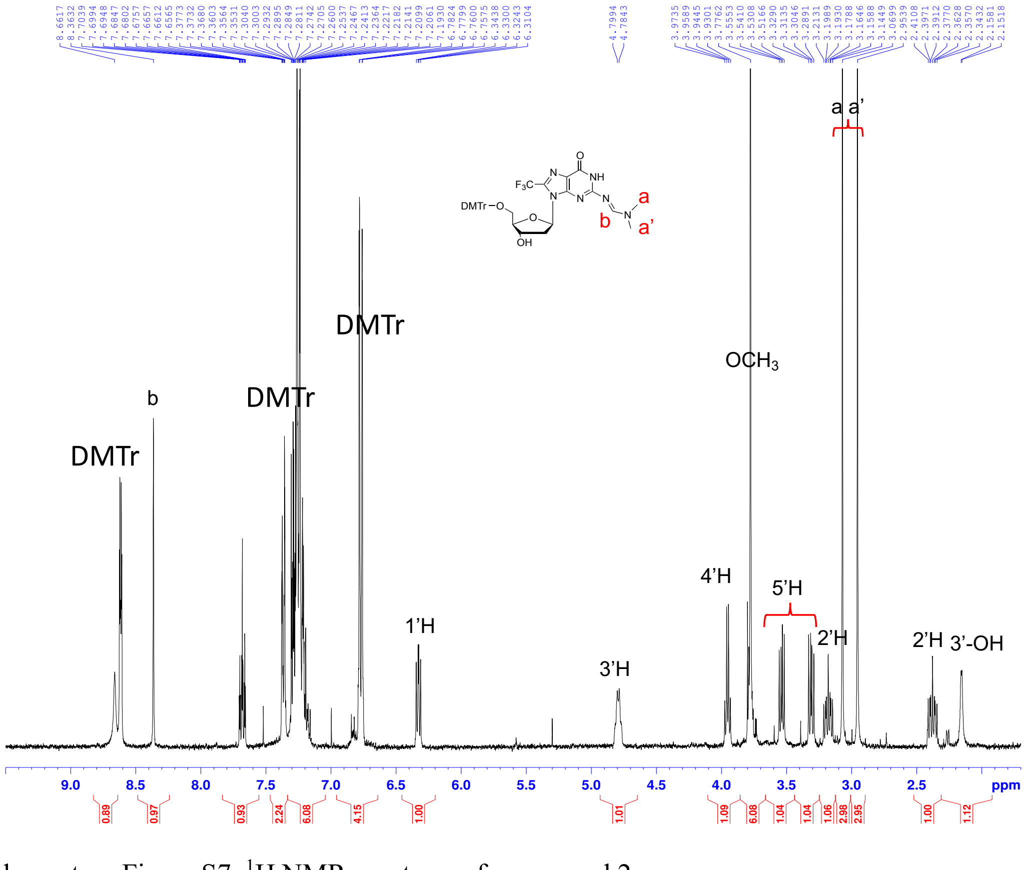

Supplementary Figure S7. ^{1}H NMR spectrum of compound 2

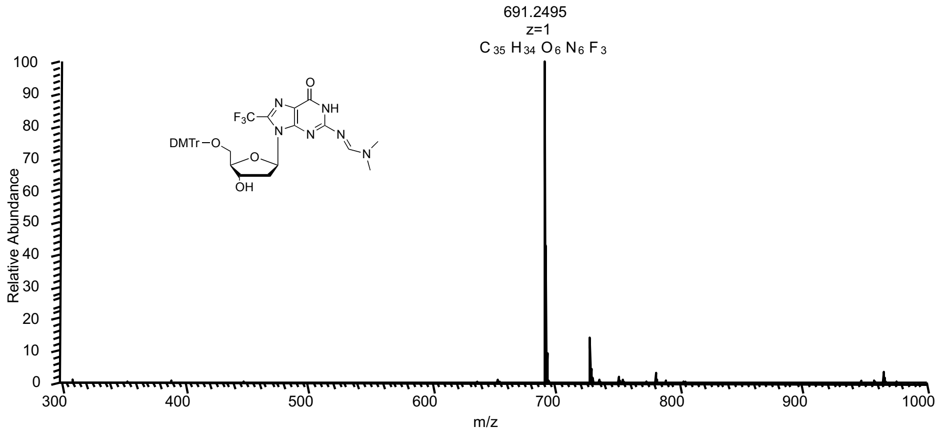

Supplementary Figure S8. HRMS spectrum of compound 2

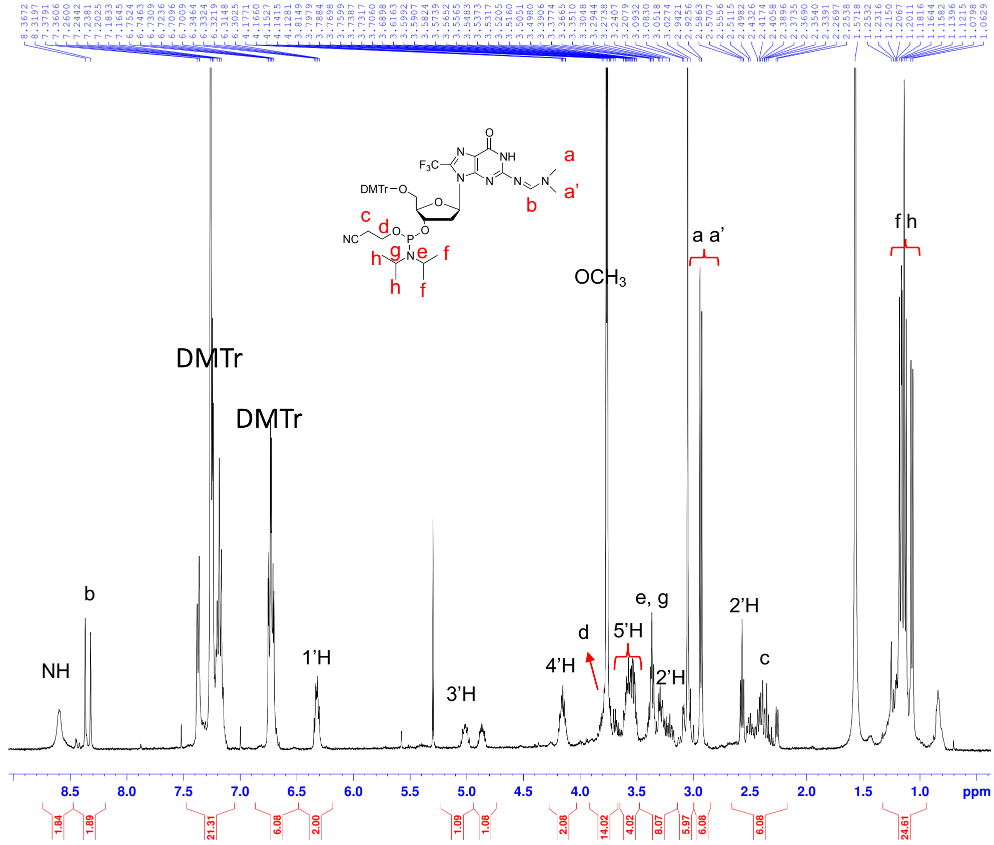

Supplementary Figure S9. ^{1}H NMR spectrum of compound 3

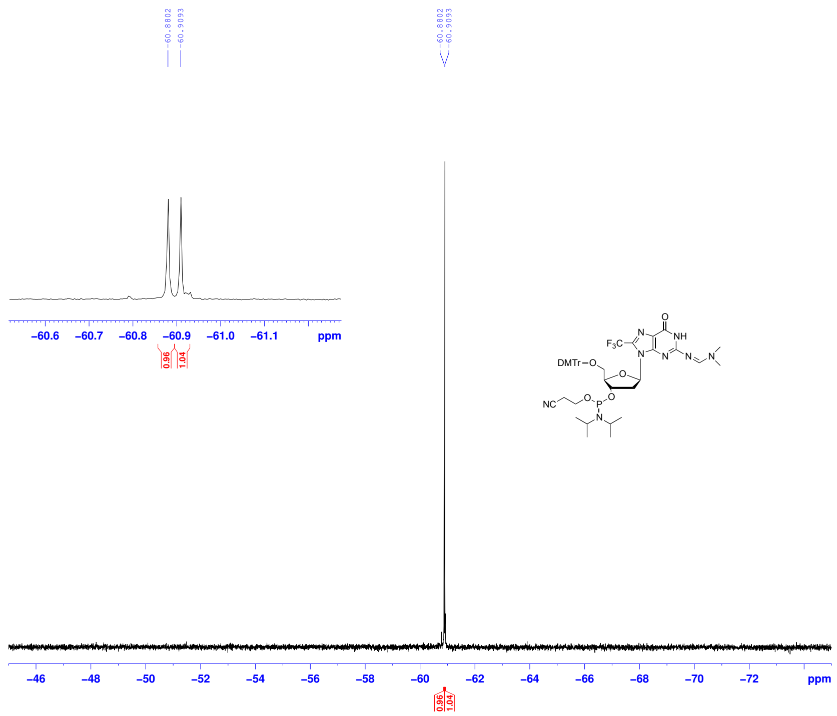

Supplementary Figure S10. ^{19}F NMR spectrum of compound 3

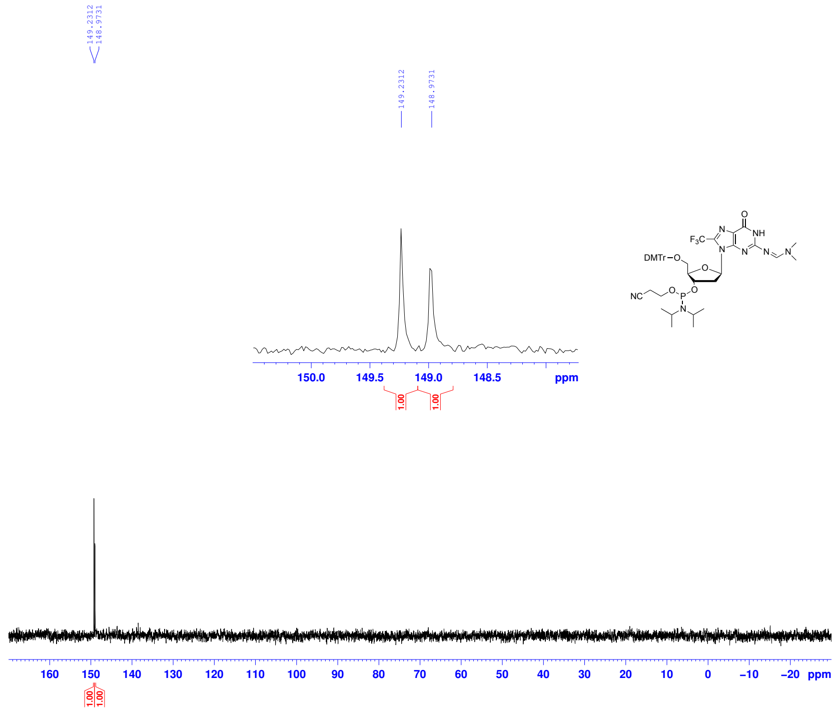

Supplementary Figure S11. ^{31}P NMR spectrum of compound 3

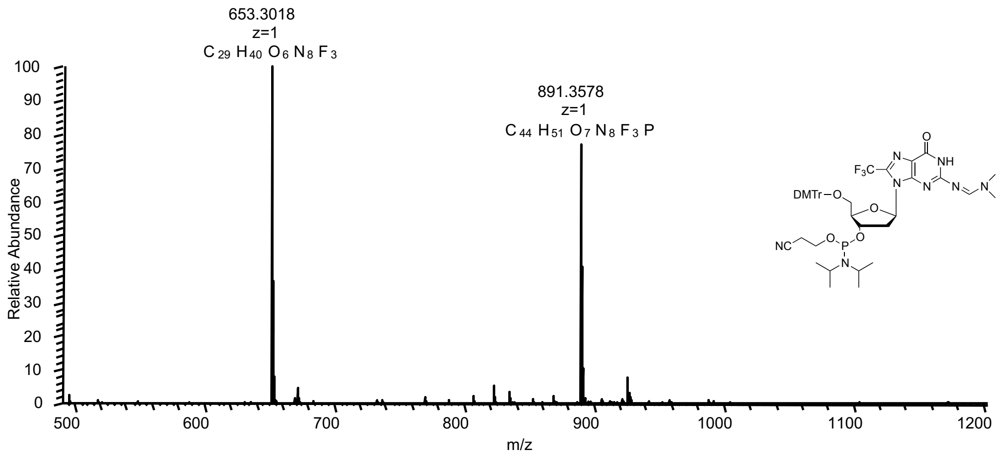

Supplementary Figure S12. HRMS spectrum of compound 3

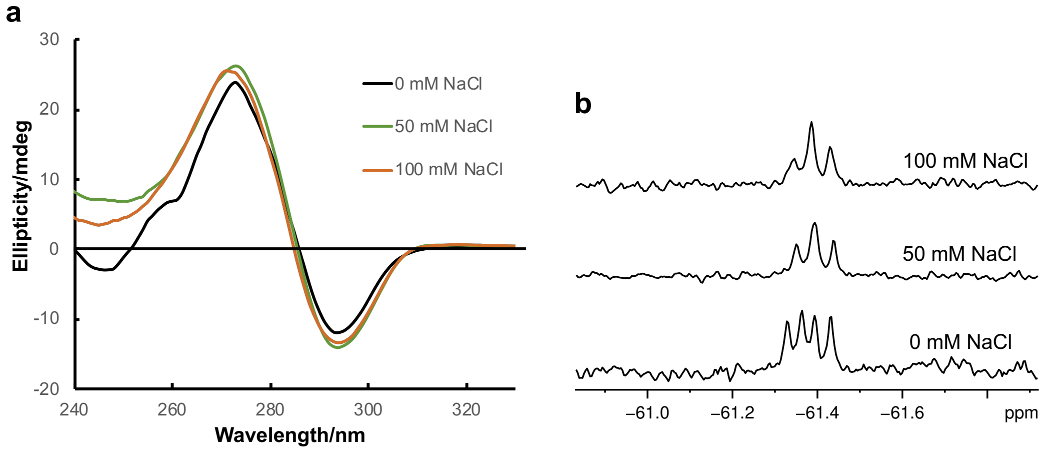

Supplementary Figure S13. a, CD spectra of d(C^{F}**G**CAC^{F}**G**CG)/d(C^{F}**G**CGT^{F}**G**CG). Condition: 15 μM duplex in 1 mM Na-PO4 buffer (pH 7.0) and various concentration of NaCl. b, ^{19}F NMR spectra of d(C^{F}**G**CAC^{F}**G**CG)/d(C^{F}**G**CGT^{F}**G**CG). Condition: 30 μM duplex in 1 mM Na-PO4 buffer (pH 7.0) and various concentration of NaCl.

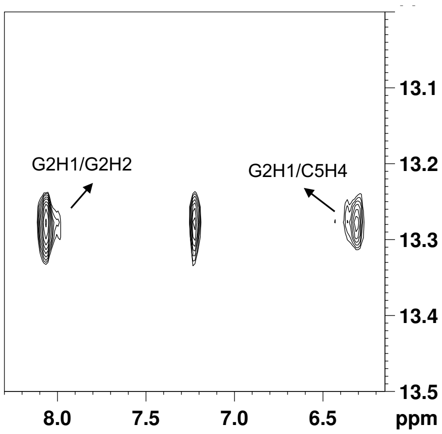

Supplementary Figure S14. The cross peaks of imino proton of G2 and amino proton of C5, indicated Watson–Crick-type base pairs of Z-DNA.

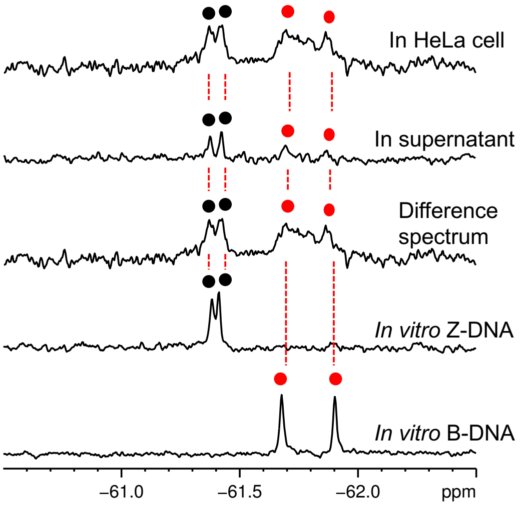

Supplementary Figure S15. In-cell ^{19}F NMR spectrum of d(C^{F}**G**CAC^{F}**G**CG)/d(CGCGTGCG). Comparison of ^{19}F NMR spectra for *in vitro* B-DNA, *in vitro* Z-DNA, in HeLa cell, in supernatant and difference spectrum between HeLa cell and supernatant.

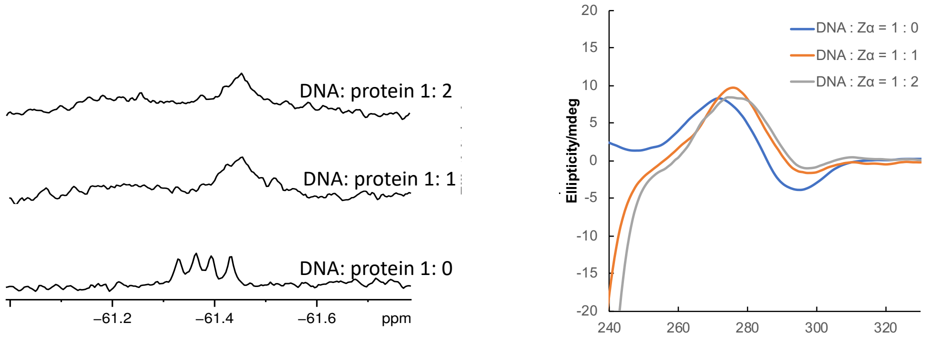

Supplementary Figure S16. ^{19}F NMR (left) spectra of four positions modified 8-mer DNA d(C^{F}**G**CAC^{F}**G**CG)/d(C^{F}**G**CGT^{F}**G**CG) and Zα protein. Condition: 30 μM DNA in 1 mM Na-PO_4 buffer (pH 7.0) and various NaCl concentrations. CD spectra (right) of four positions modified 8-mer DNA d(C^{F}**G**CAC^{F}**G**CG)/d(C^{F}**G**CGT^{F}**G**CG) and Zα protein. Condition: 5 μM DNA in 1 mM Na-PO_4 buffer (pH 7.0) and various NaCl concentrations.

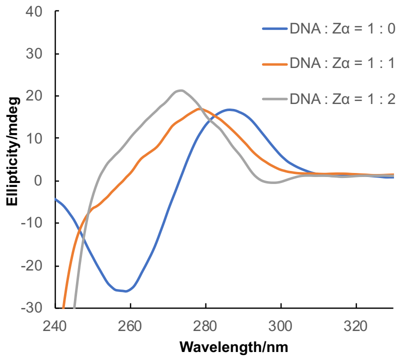

Supplementary Figure S17. CD spectra of native 8-mer DNA d(CGCACGCG)/d(CGCGTGCG) and Zα protein. Condition: 10 μM DNA in 1 mM Na-PO_4 buffer (pH 7.0) and various NaCl concentrations.

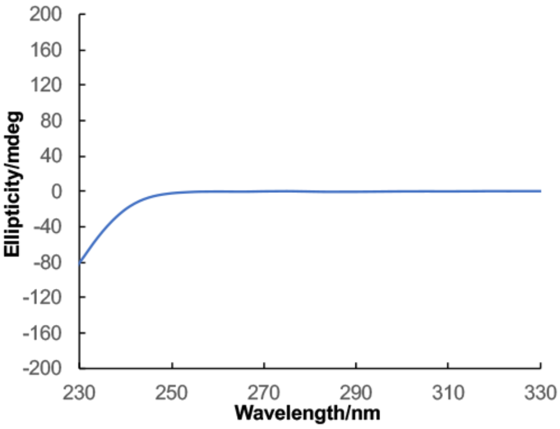

Supplementary Figure S18. CD spectrum Zα protein. Condition: 10 μM Zα protein.in 1 mM Na-PO_4 buffer (pH 7.0).

Table S1. ^{1}H NMR chemical shifts (ppm) of r(CGC^{F}GCG)$_2$ Z-DNA.

	H5	H8/6	H1'	H2'	H2''	H3'	H4'	H5'	H5''	H1	H2a/4a	H2a'/4a'
C1	5.51	7.24	5.64	1.47	2.38	4.47	3.71	2.94	3.53		6.69	6.69
G2		7.65	6.11	2.64	2.64	4.87	4.00	4.08	4.08	13.28	6.38	8.23
C3	5.03	7.24	5.64	1.59	2.51	4.70	3.71	2.41	3.71		6.30	8.07
G4			6.30	2.51	2.75	4.87	4.00	4.12	4.12	13.02	6.30	8.07
C5	5.11	7.31	5.64	1.59	2.51	4.87	3.71	2.75	4.07		6.38	8.23
G6		7.59	6.08	2.31	3.09	4.67	4.00	4.08	4.08	13.02	8.10	8.10
